# Effectiveness of Optimism Skills Group Training: Examination of the Attributional Styles of Boys at the Kerman Juvenile Correction and Rehabilitation Center

**DOI:** 10.5812/ijhrba.4412

**Published:** 2012-07-25

**Authors:** Zahra Nikmanesh, Yahya Kazemi, Mahvash Raghibi, Marjan Rabani Bavejdan

**Affiliations:** 1 Department of Psychology, Faculty of Education and Psychology, University of Sistan and Baluchestan, Zahedan, IR Iran; 2 Kerman Education Organization, Kerman, IR Iran

**Keywords:** Attribution Styles, Rehabilitation, Juvenile Delinquency

## Abstract

**Background:**

The way that people explain events may influence aspects of their development and health. A number of studies have reported that there is a positive relationship between pessimism and the risk of poor heath, infectious disease, and early mortality.

**Objectives:**

This study was conducted to examine the effectiveness of an optimism skills group training (OSGT) on the attribution styles of delinquent boys.

**Patients and Methods:**

A quasi-experimental method with a two-group design was used, with random assignment and pre- and post-tests. The subjects were selected from adolescent boys, aged 13 to 18 years, in the Kerman Juvenile Correction and Rehabilitation Center (KJCRC). The participants were allocated to two matched groups based on their pre-test scores. They were assigned randomly to the control and experimental groups. The sample comprised 61 boys. Optimism Skills Group Training (OSGT) was conducted with the experimental group during 10 sessions. Then the subjects were administered the post-test, with a follow-up test a month later. Statistical analysis was conducted using the t-test and repeated-measures analysis of variance. The research instrument was the Attributional Style Questionnaire (ASQ).

**Results:**

The results showed that the OSGT changed the attributional styles of delinquent boys from pessimistic to optimistic. Also, the follow up study showed that the effect on the delinquent boys’ attributional style was still present after one month.

**Conclusions:**

Even though the OSGT is an effective technique for inculcating an optimistic attribution style in delinquent boys, this important method needs to be continually implemented in their education.

## 1. Background

In the recent decades, attempts have been made to create a balance between studies of the negative aspects of human character and the positive aspects of human development, such as life satisfaction and strength of character (1). Positive psychology is a term for positive emotions, positive character traits, and enabling instruction (2). Seligman has also developed the concept of learned optimism. He argues that positive thinking, or optimism, results from styles of thinking about causes. These styles are called explanatory styles (3). According to these explanatory or attributional styles, the way that people explain events may influence aspects of their development and health (4). A small number of studies have been conducted on the optimistic attributional style. For example, some studies showed that people made more internal attributions for achievement than for failure, but self-esteem moderates this relationship: that is, people with high self-esteem made more external attributions for failure than those with low self-esteem. An increase in motivation can also change this relationship: when high motivation accompanied high self-esteem, people made more external attributions for failure (5). Positive psychological adjustment is indirectly associated with internal, unstable, and controllable attributions (6). Positive psychology is a scientific study of the way that positive experiences and individual character can facilitate human development. Its aim is to create a framework for happiness within three dimensions, those being a pleasant, engaged, and meaningful life (7). On the other hand, a considerable amount of research has examined how having a pessimistic attributional style can influence the symptoms of trauma (4). A pessimistic attributional style is the belief that a good event is caused by external, unstable, and specific factors and a bad event is caused by internal, stable, and global factors (8). A number of studies have investigated the relationship between pessimistic attributions and other characteristics. For example, Kamen and Seligman (8) reported that there is a positive relationship between pessimism and the risk of poor heath, infectious disease, and early mortality. Many studies have shown that pessimism is associated with clinical depression (2, 4, 9-14), and it has also been found that internal, stable, and global attributions for a specific negative event are associated with clinical depression only when the event was attributed to uncontrollable causes. Learned helplessness as a model of one type of severe clinical depression is supported in studies by Abramson, *et al*. (15). Other results have also shown that a helpless attritional style is correlated with both anxiety and depression in males (16). In addition to increasing depression, having a pessimistic attritional style predicts a decrease in physical health as well as academic and occupational achievement (8, 13, 17-21). It has been reported that subjects with the highest depression scores who had a negative attributional style for hypothetical achievement events judged such events as personally important. When they failed a mid-term exam they judged that failing the exam was of major importance. Gray, *et al*. (4) indicated that trauma-specific attributions strongly predict symptoms of posttraumatic stress disorder. In general, it is reported that stable and uncontrollable attributions are indirectly associated with negative psychological adjustment (6). Depression and depressive disorders are highly prevalent in the youth population, and therefore many treatment interventions have focused on this area (3, 9, 10, 16, 22), and have revealed that cognitive intervention can improve physical health. In the present study a behavioral and cognitive intervention was conducted to change the attritional styles of delinquent boys by using an Optimism Skills Group Training (OSGT), involving discussion, argument and homework. Therefore, this study examined the effect of the OSGT on the change of attritional style from pessimistic to optimistic in boys of the KJCRC. The research hypothesis was that the OSGT would significantly change delinquent boys’ attritional style from pessimistic to optimistic.

## 2. Objectives

The aim of present study was to examine the effect of the OSGT on the change of attributional style from pessimistic to optimistic in boys of the KJCRC.

## 3. Patients and Methods 

### 3.1. Experimental Design

The study employed an experimental method with a two-group (control and experimental) design, and pre-, post- and follow-up tests. The subjects were selected from delinquent boys who volunteered to participate in the OSGT workshop. The participants were allocated to two matched groups according to their results in the pre-test. The groups were assigned randomly to the control or experimental condition. Only the delinquent boys of the experimental group participated in the OSGT workshop, which used an optimistic teaching approach. The workshop ran for 10 sessions over 45 days. The post-test was administered to both groups two weeks after the final workshop. A follow-up test was administered to all participants one month later. The population for this research comprised adolescent boys aged 13 to 18 years in the Kerman Juvenile Correction and Rehabilitation Center (KJCRC). The sample consisted of 61 subjects who were randomly selected from 78 delinquent boys who volunteered to participate in the workshop. Sampling was conducted in several stages. First, from delinquent boys, 61 subjects volunteered to participate in the OSGT workshop. Volunteers were allocated to two matched groups according to their pre-test attributional style results: 31 subjects were assigned to the experimental group and 30 to the control group. The two groups were randomly designated as control and experimental groups. Only one participant dropped out during the workshop. Therefore the post-test and follow-up test were administered to 60 participants, 31 in the experimental group and 29 in the control group. Statistical analysis was conducted using the t-test for two independent samples. The t-test for dependent samples was also used, to compare the significant results in a repeated measures analysis of variance.

### 3.2. Research Instrument

The instrument used in this research was the Attributional Style Questionnaire (ASQ) for measuring pessimistic and optimistic attributional styles. This questionnaire was made by Peterson and Seligman (18). The ASQ consists of three attributional components, internal, stable, and global, and each component has two sections, pessimistic and optimistic (10). The ASQ has been used in several studies and has high reliability (23, 24). In this study, internal coherence estimated by the Cronbach’s alpha coefficient was 0.78.

### 3.3. An Outline of the OSGT Workshop Used in This Study

The content of the OSGT workshop was developed using Seligman, *et al*. (23) theories about the teaching of optimism. They emphasized the explanatory styles of permanence, pervasiveness, and personalization in shaping individuals’ optimistic or pessimistic characteristics. Accordingly, the purpose of the OSGT workshop was to investigate how this model can influence attribution style, changing it from pessimistic to optimistic. The guidelines for the 10 sessions of this workshop are outlined as follows:

1) In the first session, after introducing the participants in the workshop, there was a discussion about the concepts of optimism and pessimism and other characteristics of optimistic and pessimistic individuals.

2) In the second session, discussion centered on moral and behavioral characteristics of optimistic and pessimistic individuals and how these behaviors affected their lives. Two examples with pessimistic and optimistic content were presented and internal-external, global-specific, and stable-unstable attributions were emphasized.

3) In the third session, there was discussion of the differences among thinking, feeling and events, and how to recognize them. Two examples of effects on ordinary life were analyzed using the ABC model (Activating events, Beliefs and Consequences).

4) Participants were trained to use some strategies for absent-mindedness to challenge pessimistic and negative thoughts, using two examples.

5) Participants were taught how to challenge pessimistic and negative thoughts by using inconsistent beliefs and evidence, with critical discussion of two examples.

6) Anti-awfulizing was taught by explaining and critiquing two examples.

7) There was discussion of internal and external attributions. Workshop members narrated some bad memories and analyzed each one according to internal and external attributions. Participants narrated some of their unhappy memories, which were analyzed and restructured according to a different explanatory style.

8 and 9) Problem solving skills were taught, and illustrated by two examples.

10) Assertive was trained using two examples and role playing.

## 4. Results

A comparison of the pre-tests showed there was no significant difference between the two groups with respect to attribution styles (M_6_ = 14.39, M_c_ = 14.75, t = 0.34, df = 54, *P* = 0.05). This result shows that the two groups were matched at start of the study. The results of tests of the hypothesis are presented below.

### 4.1. Hypothesis

The OSGT will significantly change an attribution style from pessimistic to optimistic. A test of the homogeneity-of-slopes assumption was significant, F (2, 53) = 518.4, *P* ≤ 0.01, so the it was not appropriate to use analysis of covariance. Therefore, changes from pre-test to post-test were calculated by subtraction and the mean scores of the experimental and control groups were compared. A *t*-test, assuming equality of variances (F = 0.89, *P* ≥ 0.05), showed the following results. *Table 1* shows that the mean change score for the experimental group, M = 22.79, SD = 2.5, was significantly higher than the mean change score for the control group, M = 1.18, SD = 1.98, t (54) = 35.45, *P* ≤ .001. That is, the increase in the optimistic attribution style was significantly higher for the experimental group who participated in the workshop than for the control group. A follow-up test was conducted after 6 months. The experimental and control groups were compared using repeated-measures analysis of variance. A sphericity test showed that this method could be used. The outcomes are summarized in *Table 2*, which shows that the effect of time was significant in the experimental group, F (2, 54) = 956.04, *P* ≤ 0.01. In comparison, the effect of time was not significant in the control group, *P* ≤ 0.001 (2, 54) = 1.17, *P* ≥ 0.05. Therefore, repeated tests resulted in significantly changed scores in the experimental group. A comparison of the means and standard deviations in pre-, post-, and follow-up tests is presented in *Tabulation 1*. Since *Table 2* demonstrated that there were significant differences in the test results for the experimental group, two-by-two comparisons were conducted. A dependent t-test revealed there are significant differences among the three stages of testing. The results of these comparisons are shown in *Table 3*, which shows that the pairs were significantly different in all of the comparisons. However, there was a significant difference between the post-test and follow-up test in the second comparison, where the mean score shows a decrease in the children’s optimistic attribution style (MD = 8.86). These comparisons indicate that even though the mean score for optimistic attribution style in children decreased from the post-test to the follow-up test, more than half of the initial increase from the pre-test to the post-test (MD = 22.79) remained in the follow up test, one month later (MD = 13.93). This finding supports the research hypothesis that the experimental treatment changes attribution style from a pessimistic style to an optimistic style. This characteristic was apparent even after one month.

**Table 1.** Results Showing the Change in Optimistic Attribution Styles From Pretest to Posttest

	Mean ± SD	df	*t*- Test	*P* value
**Group**		54	35.45	0.000
Experimental	22.79 ± 2.5			
Control	1.18 ± 1.98			

**Table 2.** Results of Attribution Styles, Using the Repeat Measure Analysis of Variance

	Sum of Squares	df	Mean Square	F ^a^
**Experimental**				
Group time	7388.67	2	3694.33	0.956.04 ^b^
Error	298.66	54	3.86	
**Control**				
Group time	19.5	2	9.75	1.17 ^b^
Error	449.83	54	8.33	

^a^ Abbreviation: F, frequency

^b^ P ≤ 0.001

**Tabulation 1.** Comparing the Mean of an Optimistic Attribution Style in Three Stages

	Pretest	Posttest	Follow-up Test
Experimental, Mean ± SD	-14.39 ± 4.69	8.39 ± 3.15	-0.46 ± 2.91

**Table 3.** Results Comparing the Two by Two Tests in the Experimental Group

	Mean ± SD	df	*t*-Test	*P* value
Pretest - posttest	22.79 ± 2.54	27	47.4	0.000
Posttest-follow-up test	8.86 ± 2.26	27	20.77	0.000
Pretest - follow-up test	13.93 ± 3.41	27	21.62	0.000

## 5. Discussion

People have to learn to dispute with themselves. This strategy will help them to change their belief that everything is bad and cannot be explained. Disputation and challenge are helpful in the teaching of optimism (10). Therefore, the purpose of this research was to examine the effects of the OSGT on the optimistic attributional style. The hypothesis investigated was that the OSGT would change attributional style from pessimistic to optimistic. The results revealed that there was a significant difference between the control and experimental groups, and that the mean of the experimental group was higher than that of the control group. This means that teaching optimism by means of the OSGT enhances the optimistic attributional style. A one-month follow-up study showed that this optimistic attributional style was relatively stable. Some cognitive studies indirectly support this finding. For example, Park, *et al*. (5) reported that motivation and self esteem were related to attributional styles, Roesch and Weiner (6) found that positive psychological adjustment was associated with attributional styles. Other studies also indirectly support this finding. For example, Waschbusch, *et al*. (16) showed that a helpless attributional style correlated with anxiety. However, confirmation of a correlative relationship does not necessarily indicate a causal relationship (25). In order to establish whether a causal relationship exists, experimental or quasi-experimental research is required. Therefore, the present study used an experimental design. The results show that the OSGT is an effective method for changing an attributional style from a pessimistic style to an optimistic style in delinquent boys. These results add to the findings of Park and Peterson (1), which revealed that an optimistic attributional style can improve people’s wellbeing and health. These findings also show that the optimistic attributional style in delinquent boys remained present after one month. However, when the OSGT ceased, there was a relative decrease in its positive effects. Therefore, even though the OSGT is an effective technique for improving an optimistic attributional style in delinquent boys, this important method needs to be continually implemented in their education. In this way, delinquent boys can continue to experience an optimistic attributional style.
